# Effect of Addition Amount of Microbial Self-Repairing Material on Anti-Cracking Performance of Concrete

**DOI:** 10.3390/ma19122540

**Published:** 2026-06-12

**Authors:** Hai-Yan Zhang, Hu-Bin Bai, Gui-Qiang Li, Yu-Jiao Zhang, Hui Rong, Xiang-Guo Li

**Affiliations:** 1 China Testing & Certification International Group Xi’an Co., Ltd., Xi’an 710061, China; zhy0921a@163.com (H.-Y.Z.);; 2 School of Materials Science and Engineering, Wuhan University of Technology, Wuhan 430070, China; 3 College of Soil and Water Conservation Science and Engineering, Northwest A&F University, Xianyang 712100, China; 4 School of Materials Science and Engineering, Tianjin Chengjian University, Tianjin 300384, China; 5 Joint Research Center for Chinese and Polish Building Green Functional Materials and Technology, Tianjin Chengjian University, Tianjin 300384, China; 6 Tianjin Key Laboratory of Functional Materials for Building Green Materials, Tianjin 300384, China

**Keywords:** concrete, microbial remediation, plate cracking, self-healing effect, microscopic analysis

## Abstract

Although microbial self-healing concrete technology has been widely studied, limited attention has been paid to the effect of the dosage of microbial self-healing materials on concrete crack repair performance. To address this, this study investigates the influence of the dosage of microbial self-healing materials on the crack repair performance of concrete using planar thin-plate specimens. The results are summarized as follows: (1) Increasing the dosage of microbial self-healing materials effectively delays the initial cracking time of concrete specimens. When the dosage levels were 10%, 20%, and 30%, the initial cracking time was prolonged by 50%, 65%, and 70%, respectively, compared with the blank group without microbial addition. (2) After 28 d of water spraying and coating curing, the total crack area of concrete decreased significantly compared with that at the early age (1 d). For dosages of 0%, 10%, 20%, and 30% of microbial self-healing materials, the total crack area per unit surface area decreased by 12.2%, 21.9%, 22.7%, and 31.8%, respectively, compared with the initial stage. (3) Through X-ray diffraction (XRD), thermogravimetric analysis (TG/DTG), and morphological characterization, the presence of microbial mineralization products, including calcite and vaterite, on the concrete crack surfaces was confirmed.

## 1. Introduction

The widespread application of concrete has brought many conveniences to people; however, during use, concrete components often develop various types of cracks [1]. Among them, 80% of concrete cracks are caused by non-load-related factors, especially shrinkage cracks. Therefore, it is necessary to repair non-load-induced cracks [2], especially early-stage shrinkage cracks, which is of great significance for mitigating the deterioration in the durability of concrete structures caused by crack expansion [3,4,5].

At present, concrete crack repair methods mainly include surface treatment [6], grouting [7], concrete replacement [8], and structural reinforcement [9]. However, most of these methods are passive or post-repair measures, which often lead to a large waste of manpower and material resources [10]. To address the shortcomings of conventional repair methods, microbial self-healing technology has emerged as a potential approach for effective crack repair. Compared with conventional repair technologies, microbial self-healing technology repairs concrete cracks or pores through calcium carbonate produced by microbial mineralization and has the advantage of automatic repair [11,12,13]. However, although microbial self-healing technology has been increasingly studied for concrete crack repair [14,15,16,17,18], research on the effect of the amount of microbial self-healing material used on concrete crack repair performance remains insufficient [19].

In recent years, a series of breakthroughs have been made in studies on the mechanisms of microbial self-healing [20,21,22]. The “two repairs” theory proposed by Jonkers’ team [23] includes a dynamic repair mechanism: the initial repair stage (0–60 days) achieves initial crack sealing through the rapid deposition of CaCO_3_, whereas the secondary repair stage uses wet–dry cycles to activate dormant spores and repair deep cracks for more than 5 years. Wiktor et al. [24] used X-ray tomography technology to find that 97% of CaCO_3_ deposition is concentrated in the shallow layer of the fracture (within 0.2 mm), verifying the necessity of multiple repairs. Regarding the issue of the amount of microbial self-healing materials, Xu et al. [25] confirmed through a systematic dosage test (0.5–5%) that a dosage of 1.5–2.5% can achieve a crack closure rate of 60–80%; however, when the dosage exceeds 3%, the compressive strength decreases by 10–15% due to carrier pores. The microbial + nano-SiO_2_ composite system developed by Zhang et al. [26] (0.8% dosage) repaired 40% of the early-stage crack area without affecting the final strength. Seifan et al. [27] verified that at a dosage of 2% under marine conditions, the protective effect against the chloride ion diffusion coefficient reached 65%. The freeze–thaw cycle test conducted by Li et al. [28] showed that the specimen with a 2% dosage still exhibited a 70% repair rate after 300 freeze–thaw cycles.

However, existing studies still lack sufficient information on the negative effects associated with high dosages, particularly the absence of a coordinated evaluation system for early-age crack resistance and later-age crack repair. Therefore, this study employs planar thin-plate molds to induce early-age shrinkage cracks in concrete and investigates the effects of microbial self-healing materials on early-age (1 d) crack resistance and later-age (28 d) crack repair performance. To establish a more systematic evaluation framework, X-ray diffraction (XRD), thermogravimetric analysis (TG/DTG), and scanning electron microscopy (SEM) were employed to characterize the microstructural evolution and healing products of concrete containing microbial self-healing materials, thereby improving the coordinated evaluation of early-age crack resistance and later-age repairability.

## 2. Testing

### 2.1. Raw Materials

Cement: P·O 42.5 ordinary Portland cement was supplied by Tangshan Jinma Qixin Cement Co., Ltd. (Tangshan, China), with a specific surface area of 421 m^2^/kg. The chemical composition of the cement was provided by the manufacturer, and its main oxide components are listed in Table 1. Based on these data, the mineral composition of the cement clinker was estimated using the Bogue calculation method, indicating that the principal clinker phases were C_3_S, C_2_S, C_3_A, and C_4_AF (Table 2). Fine aggregate: River sand with a fineness modulus of 2.8 and an average particle size of 0.35–0.50 mm was used. Coarse aggregate: Crushed gravel with a particle size ranging from 5 to 25 mm was used. Fly ash: Grade II fly ash, whose chemical composition is presented in Table 3, was used as provided by the manufacturer. Water-reducing agent: Polycarboxylate-based high-performance water-reducing agent was used. Water: Tap water was used unless otherwise specified. Microbial self-healing material: The microbial self-healing material consisted of Component A and Component B. Component A was a Pasteurella solution with a microbial concentration of approximately 10^9^ cells/mL. Component B consisted of urea and calcium acetate.

### 2.2. Experimental Program

#### 2.2.1. Concrete Mix Ratio

This study investigated the effects of Component A of the microbial self-healing material on the early-age (1 d) crack resistance and later-age (28 d) crack-healing performance of concrete by varying its dosage. The detailed mix proportions are presented in Table 4. Group A served as the control group without any microbial self-healing material. Group B consisted of specimens containing only Component A, while Group C consisted of specimens containing only Component B. Groups D, E, and F contained both Component A and Component B, with Component A replacing 10%, 20%, and 30% of the mixing water, respectively. These groups were designed to evaluate the influence of microbial self-healing material dosage on the early-age crack resistance and later-age crack-healing performance of concrete. Furthermore, the healing mechanism was analyzed through the macroscopic morphological evolution before and after crack repair, crack-healing rate measurements, and microstructural characterization of the healing products formed within the cracks.

#### 2.2.2. Test Preparation Process

According to the GB/T 50082-2009 [29], concrete specimens were prepared, and a crack inducer was embedded to create a stress concentration zone. Combined with controlled airflow to accelerate surface moisture evaporation, shrinkage cracks were induced in the stress concentration zone to evaluate the early-age cracking performance of microbial self-healing concrete.

**Figure 1:**
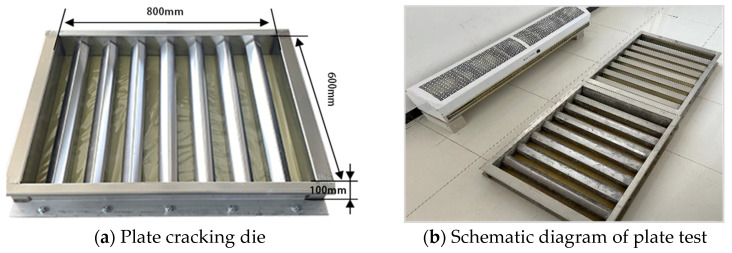
Plate cracking die and test diagram.

(1) Thirty minutes after casting, the flat concrete specimens were placed in front of an air curtain with an adjusted airflow. The wind speed measured at 100 mm above the center of the specimen surface was maintained at (5 ± 0.5) m/s. The ambient temperature and relative humidity were controlled at (28 ± 2) °C and (70 ± 5)%, respectively. The airflow direction was kept parallel to both the specimen surface and the crack inducer, as shown in Figure 1. (2) The test duration was calculated from the time of water addition during concrete mixing. After (24.0 ± 0.5) h, crack widths were measured using a steel ruler and a 40× reading microscope (Model 10085-2, Beijing Haichuang Gaoke Technology Co., Ltd., Beijing, China). During the test period, the time of crack initiation and the crack development process were continuously observed and recorded.

### 2.3. Test Methods

(1) Statistics of crack macroscopic morphology: A 20-megapixel camera was used to capture images of the cracks on the concrete surface before and after repair, enabling a direct visual evaluation of the repair effect. Macroscopic parameters, including crack size, number of cracks, and initial cracking time of the concrete slab specimens, were recorded. Based on these data, the variation trends in the initial cracking time of each group, as well as the changes in crack area and crack number before and after repair, were calculated and plotted.

(2) Crack area repair rate: Crack widths were measured using a 40× reading microscope, and images of the cracks were captured at both 1 d and 28 d. Based on the recorded crack evolution, the variation in crack area between 1 d and 28 d was determined. The crack area repair rate (μ), representing the degree of crack closure after 28 days of healing, was calculated according to the changes in crack area before and after repair.

(1) $$\mathrm{Area}\;\mathrm{repair}\;\mathrm{rate}:\;\mu =\frac{S_{1d}-S_{28d}}{S_{1d}}$$

(2) $$\mathrm{Total}\;\mathrm{crack}\;\mathrm{area}:\;S_{nd}=\frac{1}{2}\sum_{i}^{m}\left(W_{i}\times L_{i}\right)$$

where S_nd_ is the total crack area after n days of repair, mm^2^; S_1d_ is the initial total crack area at 1 d, mm^2^; S_28d_ is the total crack area after 28 d of repair, mm^2^; W_i_ is the maximum width of the i-th crack, mm; L_i_ is the length of the i-th crack, mm; and m is the total number of cracks.

**Figure 2:**
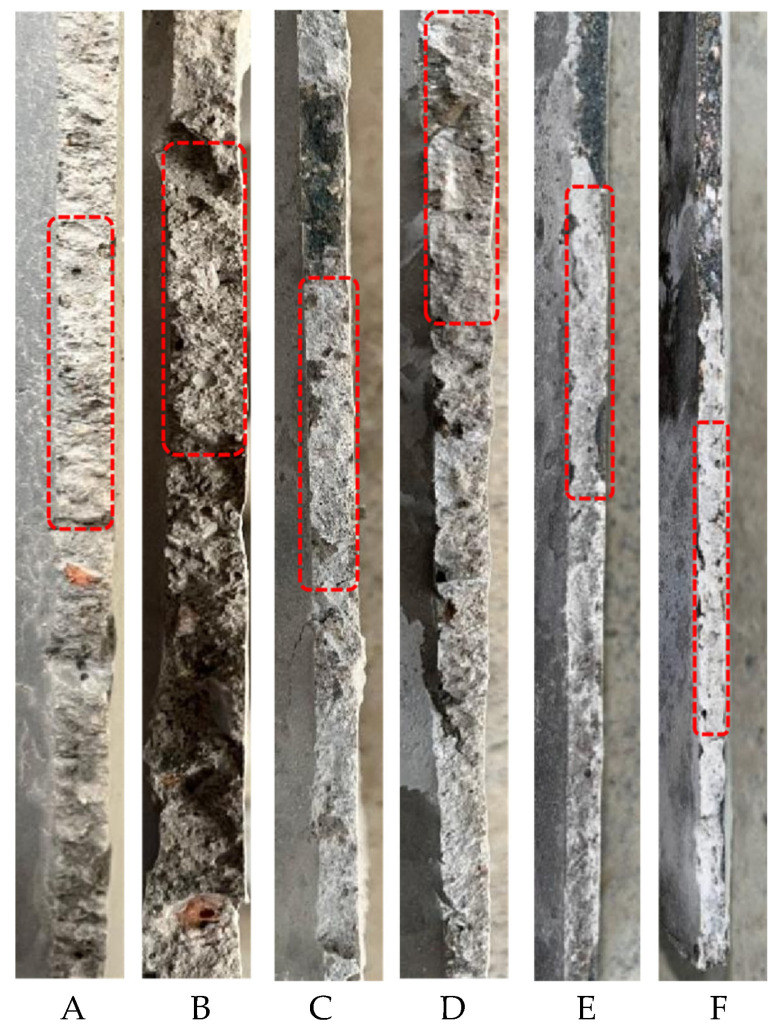
Fracture surface and sampling points of each group. (**A**) Group A; (**B**) Group B; (**C**) Group C; (**D**) Group D; (**E**) Group E; and (**F**) Group F. The red dashed frames indicate the sampling locations selected for subsequent analysis.

(3) Microscopic characterization of crack-healing products: After 28 d of healing, the microbial self-healing concrete slab specimens were crushed, and the concrete fragments containing the healed crack surfaces were collected (Figure 2). The surfaces of the specimens were carefully polished using a file prior to testing. First, phase analysis of the concrete powder samples was conducted using an X-ray diffractometer (XRD, JN-210, Rigaku, Tokyo, Japan). The scanning range of 2θ was 5–85°, and the scanning rate was 6°/min. Second, thermogravimetric analysis was performed using a Q600 simultaneous thermal analyzer (TA Instruments, New Castle, DE, USA). The samples were heated from room temperature to 800 °C under a nitrogen atmosphere at a heating rate of 20 °C/min, and the mass loss and corresponding thermal changes were recorded throughout the test. Finally, the concrete fragments containing the healed crack surfaces were examined using a JSM-7800F scanning electron microscope (JEOL Ltd., Akishima, Tokyo, Japan). After vacuum treatment, the specimens were observed under an accelerating voltage of 5–15 kV for microstructural characterization and comparison.

## 3. Test Results and Discussion

Under relatively stable environmental conditions, including a wind speed of (5.0 ± 0.5) m/s, a relative humidity of (70 ± 5)%, and a temperature of (28 ± 2) °C, the crack size, crack number, and initial cracking time of the specimens were measured 24 h after casting. The obtained results are summarized in Table 5. The healing performance of early-age cracks was investigated through water spraying and film curing. The influence of microbial self-healing material dosage on crack healing was evaluated by comparing the changes in crack size and crack number before and after healing. In addition, the crack-healing conditions of the specimens at 1 d and 28 d under different dosages of microbial self-healing materials are presented in Figure 2.

### 3.1. Initial Cracking Time of Concrete

**Figure 3:**
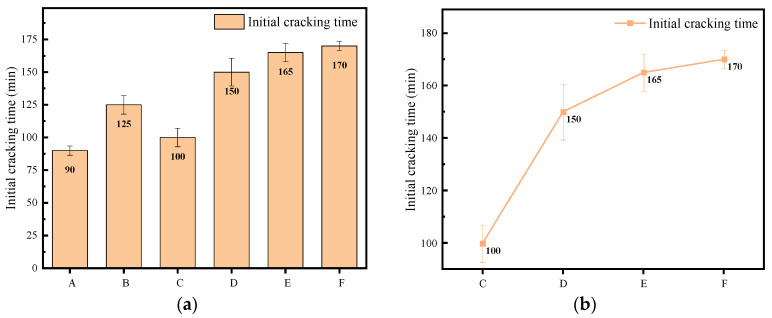
Initial cracking time of each concrete slab. (**a**) Initial cracking time of different concrete slabs; (**b**) Effect of microbial self-healing material component a content on the initial cracking time.

The initial cracking times of the concrete slabs are presented in Figure 3. As shown, Group A exhibited the earliest initial cracking time of (90 ± 5) min, followed by Group C at (100 ± 5) min and Group B at (125 ± 5) min. For Groups D, E, and F, the initial cracking time increased progressively with the dosage of Component A of the microbial self-healing material (10%, 20%, and 30%), reaching (150 ± 5) min, (165 ± 5) min, and (170 ± 5) min, respectively. The difference in initial cracking time between Groups A and B can be mainly attributed to the organic nutrients contained in Component A of the microbial self-healing material, including beef extract, urea, and tryptone used during the bacterial cultivation process. These substances can form a film on the surface of cement particles, thereby reducing the hydration rate of cement and delaying the onset of cracking. For Group C, which contained only Component B of the microbial self-healing material, the initial cracking time was delayed by approximately 10 min compared with that of Group A. This is because calcium acetate and urea can jointly form a thin layer of white deposits on the concrete surface, partially blocking the direct pathways between the concrete and the external environment, thereby slowing moisture evaporation and delaying the hydration process. In addition, the highly alkaline environment generated during cement hydration is generally unfavorable for bacterial growth. However, the degradation of organic nutrient substrates produces acidic intermediates that can partially neutralize and buffer the alkalinity of the cement matrix. This creates a relatively mild microenvironment for Pasteurella, promoting its survival and enabling the induction of calcium carbonate precipitation for crack healing. When Components A and B were incorporated simultaneously, the initial cracking time was further prolonged due to their combined effects. Compared with Group A, the initial cracking times of Groups D, E, and F increased by 66.7%, 83.3%, and 88.9%, respectively.

The relationship between the dosage of Component A of the microbial self-healing material and the initial cracking time of concrete is presented in Figure 3. As shown, the initial cracking time increased with the dosage of Component A; however, the rate of increase gradually decreased, as reflected by the declining slope (k) of the fitted curve. For Groups C, D, E, and F, in which the dosage of Component A was controlled, the initial cracking times of Groups D, E, and F increased by 50%, 65%, and 70%, respectively, compared with Group C, which did not contain Component A. The initial cracking time of Group C was approximately 100 min, whereas that of Group F, with the highest dosage of Component A (30%), reached approximately 170 min, corresponding to an increase of about 70%. This phenomenon can be attributed to the increasing amount of organic nutrients and microbial metabolites with retarding effects introduced by Component A as its dosage increased from 0% to 10%, 20%, and 30%. These substances gradually enhanced the retardation of cement hydration, thereby prolonging the initial cracking time of concrete. However, the magnitude of this effect gradually diminished at higher dosages, resulting in a decreasing growth rate of the initial cracking time and a tendency toward stabilization. This may be related to the progressive coverage of cement particle surfaces by organic substances, after which additional increases in dosage contribute less significantly to the retardation effect.

### 3.2. Crack Changes Before and After Concrete Crack Repair

Based on the theory of microbially induced calcium carbonate precipitation (MICP), the formation of cracks in concrete allows the ingress of moisture and oxygen, thereby activating dormant Pasteurella within the cracks. The revived bacteria secrete urease, which catalyzes the hydrolysis of urea to generate CO_3_^2−^. Meanwhile, the negatively charged bacterial cell walls serve as nucleation sites for the adsorption of Ca^2+^, resulting in the in situ precipitation of stable calcite crystals within the cracks and thereby promoting crack healing.

**Figure 4:**
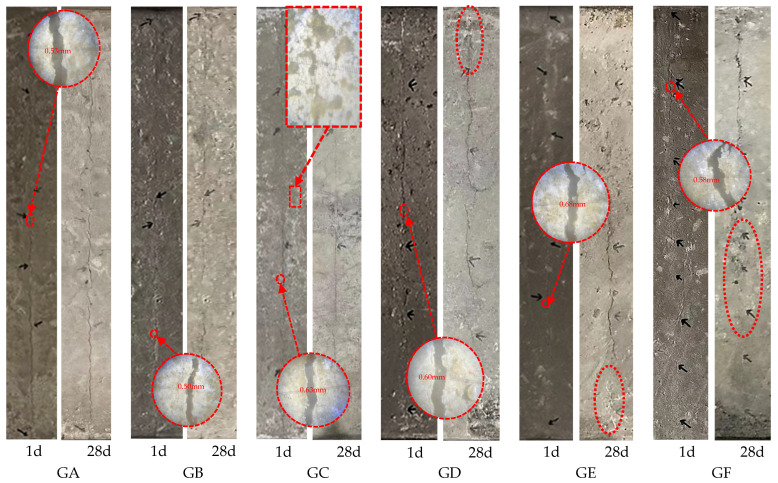
Fracture repair of samples in each group at 1 d and 28 d.

**Figure 5:**
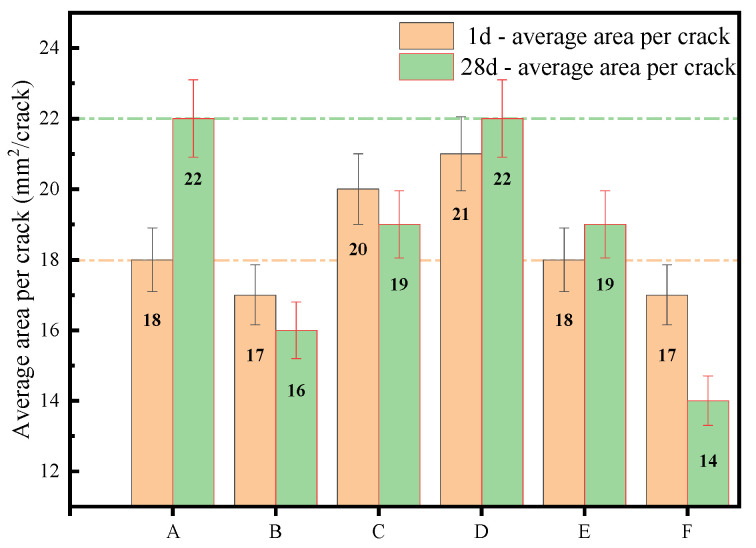
Variation in the average area of each crack in each group of concrete.

**Figure 6:**
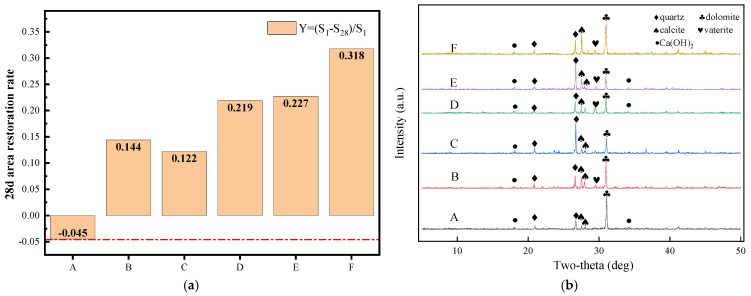
(**a**) The change in the 28 d area repair rate of concrete cracks. (**b**) XRD patterns of fracture surface samples of each group.

**Figure 7:**
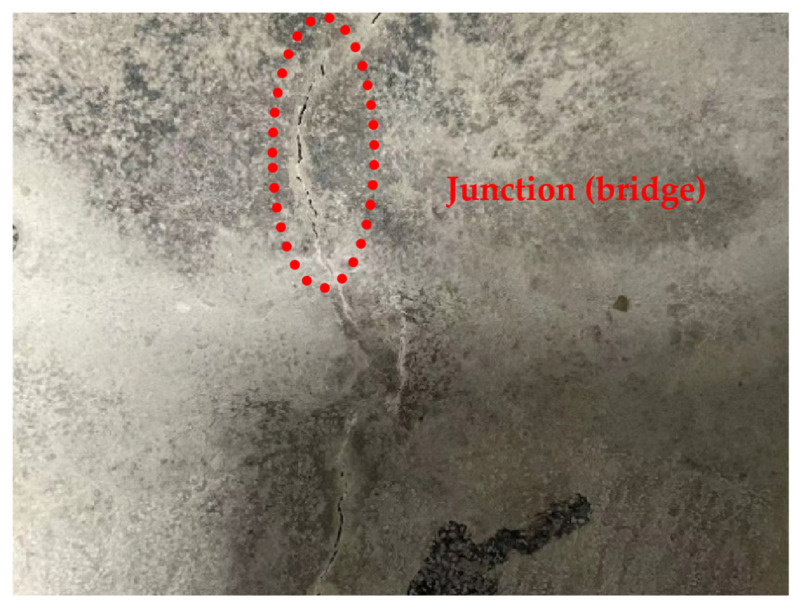
Repair junction at the crack (bridge).

**Figure 8:**
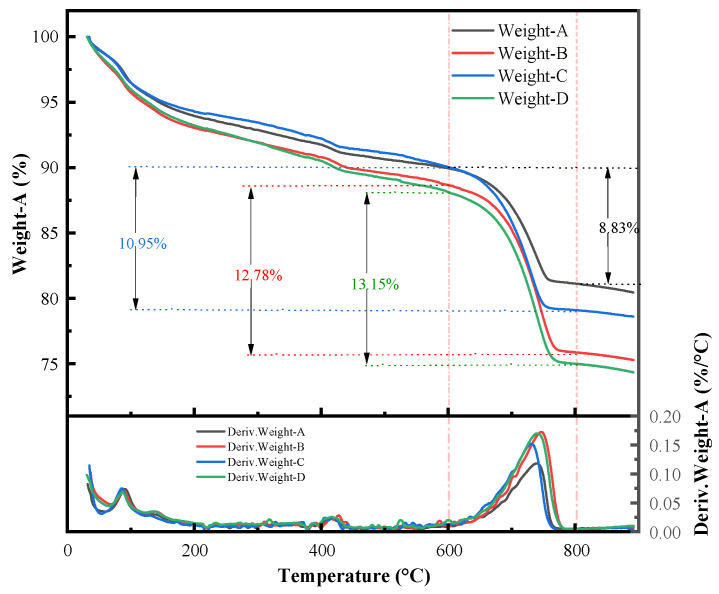
Thermal analysis of ABCD group samples.

**Figure 9:**
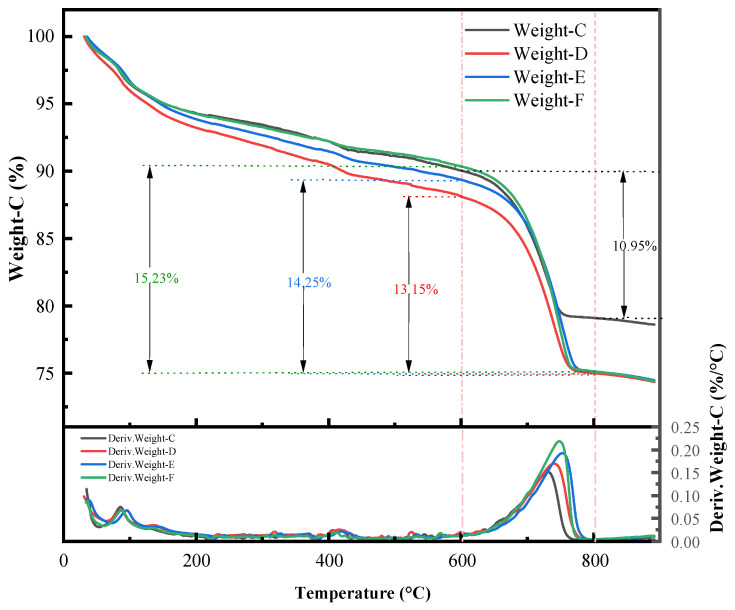
Thermal analysis of CDEF group samples.

The crack patterns of concrete slabs at 1 d and after 28 d of healing under different dosages of microbial self-healing materials are presented in Figure 4, Figure 5, Figure 6, Figure 7, Figure 8 and Figure 9. It was observed that after 1 d of induced cracking, Group F, in which Component A accounted for 30% of the microbial self-healing material, exhibited the largest number of cracks, although the individual crack lengths were relatively short. In contrast, when only Component B was incorporated (Group C), the maximum crack length reached 221 mm. Among all groups, the control group (Group A) exhibited the smallest number of cracks, with only 28 cracks recorded. As the proportion of Component A increased, the maximum crack length gradually decreased, whereas the number of cracks gradually increased. After 28 d of water spraying and film curing, Group F exhibited the most pronounced crack-healing effect, followed by Groups E and D. In contrast, compared with the crack condition observed at 1 d, no significant changes were observed in the control group (Group A) after 28 d of curing.

#### 3.2.1. Average Crack Area

The variation in the average area per crack for each group is presented in Figure 5. The results indicate that all components of the microbial self-healing material influenced the average crack area of concrete. Among the tested groups, Group B, which contained only Component A of the microbial self-healing material, exhibited the smallest average crack area (17.0 mm^2^). However, the increased number of cracks resulted in a larger total crack area per unit surface area in this group. For Groups C, D, E, and F, the average crack area showed a changing trend with the dosage of Component A. When the dosage of Component A was 10% (Group D), the average crack area reached the maximum value of approximately 21 mm^2^. As the dosage of Component A increased further, the average crack area gradually decreased. After 28 d of healing, the average crack area in Groups A, C, and D changed for different reasons. In Group A, the increase in the average crack area was mainly attributed to the continued development of plastic shrinkage cracks after the initial cracking stage (1 d). As a result, numerous short cracks gradually merged and developed into fewer but longer and wider cracks. This phenomenon was also reflected by the decrease in crack number per unit area. In contrast, the opposite trend was observed in Groups C and D. Under the action of the microbial self-healing materials, many fine cracks were partially or completely healed, resulting in a substantial reduction in crack number while producing only a limited change in the total crack area. Consequently, the average crack area per remaining crack increased.

#### 3.2.2. Area Restoration Rate

The crack areas at 1 d and 28 d were recorded based on the same observations. By calculating the difference between the crack areas at 1 d and 28 d, the 28 d crack-healing rates of concrete containing different microbial self-healing materials were obtained and are presented in Figure 6a. As shown in Figure 6a, the concrete specimens containing different components of the microbial self-healing materials exhibited varying degrees of crack-healing performance. For Groups A, B, C, and D, the specimen containing both Component A and Component B (Group D) exhibited a significantly improved healing effect compared with the specimens containing only one component. The crack-healing rate of Group D was 52.1% and 44.3% higher than that of the corresponding single-component groups, indicating a synergistic effect between Components A and B. For Groups C, D, E, and F, the crack-healing rate gradually increased with the dosage of Component A of the microbial self-healing material (0–30%). The maximum crack-healing rate reached 31.8%, demonstrating that increasing the dosage of Component A enhanced the crack-healing capability of concrete within the investigated dosage range.

### 3.3. Concrete Crack Surface Microscopy

#### 3.3.1. X-Ray Diffraction

After 28 d of healing, the concrete slab specimens were crushed, and concrete samples from the crack surfaces were collected for microscopic characterization, as shown in Figure 6b. First, X-ray diffraction (XRD) analysis was performed on the powdered samples obtained from the crack surfaces. The phase composition corresponding to the diffraction peaks was identified by comparison with standard PDF cards, following established procedures for phase analysis of cement-based materials reported in previous studies [30,31].

The XRD results indicate that the crack surface is mainly composed of calcite (CaCO_3_, PDF# 05-0586), quartz (SiO_2_, PDF# 46-1045), dolomite (CaMg(CO_3_)_2_, PDF #36-0426), vaterite (CaCO_3_, PDF #33-0268), and portlandite (Ca(OH)_2_, PDF #44-1481), suggesting the coexistence of multiple mineral phases on the crack surface. Dolomite and quartz originate from the aggregates used in the experiment and are inherent inert phases of the raw materials; portlandite (Ca(OH)_2_) is a major product of the hydration of silicate cement. Under microbial mineralization, the newly formed minerals in the system are predominantly calcium carbonate polymorphs, specifically metastable vaterite and thermodynamically stable calcite. These two phases are the principal products of microbially induced calcium carbonate precipitation (MICP), playing key roles in crack filling, particle binding, and structural reinforcement. Based on the diffraction peak intensities, the characteristic peaks of vaterite are relatively weak, whereas the main peak of calcite is more pronounced. This indicates that most of the metastable vaterite has undergone recrystallization into the more thermodynamically stable calcite, which is consistent with findings reported in previous studies [32].

The concrete at the crack surface contains repair products of microorganisms, but the repair products of microorganisms are difficult to be aggregated or fixed at the cracks; this is mainly because cracks in this study were induced by forced air drying and rapid shrinkage, resulting in a high wind speed and accelerated moisture evaporation in the test environment. Meanwhile, the early-age cracks generated under slab knife-edge restraint exhibit rough internal surfaces and irregular morphologies. The combined effects of these environmental conditions and crack characteristics make it difficult for microbial mineralization products to form effective interlinked (bridging) structures within the cracks (see Figure 7). In contrast, the formation of calcium carbonate bridging structures through microbial mineralization typically requires a relatively closed, moist, and stable environment. Therefore, the microbial crack-healing efficiency in concrete is limited under the present test conditions.

#### 3.3.2. Heat

The powder of the repair product sample was heated (TG/DTG) (as shown in Figure 8 and Figure 9). Based on relevant literature, it was learned that calcium carbonate would be decomposed by heat within the temperature range of 600–800 °C. The amount of heat decomposition of the sample is directly related to the sampling and the amount of bacterial fluid, so it can only supplement and prove the main substances calcite and vaterite on the concrete crack surface.

As shown in Figure 8, the thermogravimetric (TG/DTG) curves of samples A, B, C, and D were analyzed to evaluate the effect of different components of the microbial self-healing materials on crack healing. It can be observed that the control group (Group A), without any microbial self-healing material, exhibited the smallest mass loss in the temperature range of 600–800 °C, accounting for only 8.83%. In comparison, the mass losses in Groups B, C, and D were 12.78%, 10.95%, and 13.15%, respectively. For Group B, which contained only Component A of the microbial self-healing material, the observed increase in mass loss indicates the formation of calcium carbonate products in the concrete, even without the direct addition of an external calcium source. This can be attributed to the presence of free Ca^2+^ ions generated during the cement hydration process, which provide the necessary ions for microbial-induced mineralization. Compared with Group B, the mass loss of Group D, which contained both Component A and Component B, increased by only 0.37%. This suggests that the additional contribution of Component B to calcium carbonate formation is limited under conditions with small and micro-scale cracks. Therefore, when only minor cracks are present, the addition of Component A alone may be sufficient to achieve effective crack-healing performance.

As shown in Figure 9, the thermogravimetric (TG/DTG) results of concrete samples (Groups C, D, E, and F) with different dosages of Component A of the microbial self-healing material were analyzed. It can be observed that Group C, which did not contain Component A, exhibited a mass loss of only 10.95% in the temperature range of 600–800 °C, whereas Groups D, E, and F, with Component A dosages of 10%, 20%, and 30%, showed increased mass losses ranging from 13.15% to 15.23%. By analyzing the mass loss within the 600–800 °C range (corresponding to the decomposition of CaCO_3_) and comparing it with the crack area healing ratio, it was found that with increasing dosage of the microbial self-healing material, the estimated CaCO_3_ content increased from approximately 10.95% to 15.23%. Meanwhile, the crack area healing ratio also increased significantly, reaching up to 31.8%. These results indicate that the improvement in crack-healing performance is closely related to the increased formation of calcium carbonate, suggesting a positive correlation between CaCO_3_ precipitation and crack-healing efficiency.

#### 3.3.3. Morphological Analysis

**Figure 10:**
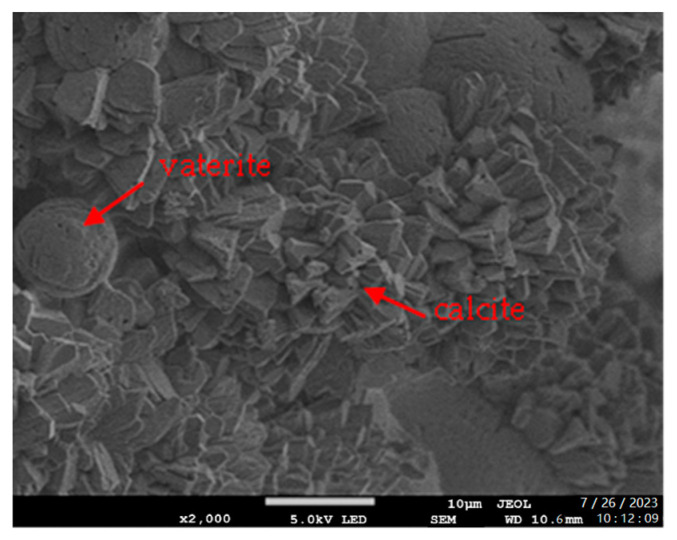
Laboratory microbial mineralizations.

**Figure 11:**
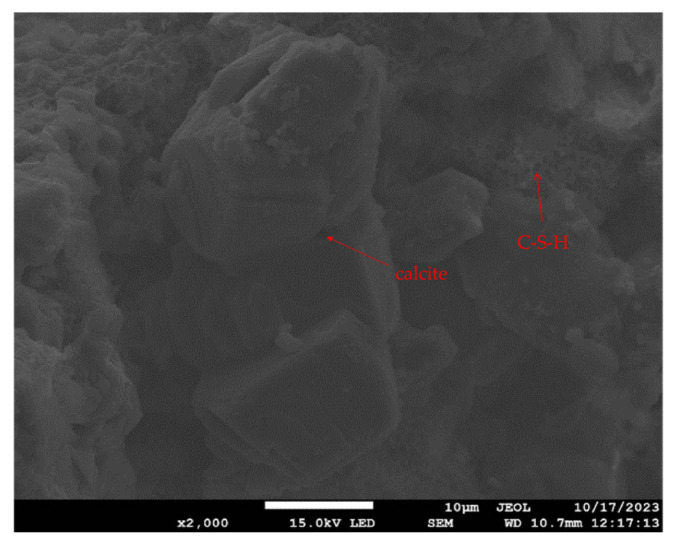
Calcite on the crack surface of concrete.

Through scanning electron microscopy (SEM) observation of the crack surfaces (Figure 10 and Figure 11), regions without distinct crystal boundaries and with a relatively loose microstructure were identified, which are typically associated with low-crystallinity phases and are therefore likely attributed to C–S–H gel. In addition, the crack regions were covered by a large number of micron-sized blocky and flaky deposits, forming an overall relatively dense packing structure. Some of these deposits exhibited well-defined crystalline morphologies, which can be identified as calcite crystals. Meanwhile, a small amount of agglomerated or irregularly clustered particles was also observed, whose morphology shows certain similarities to that of vaterite. These observations are consistent with those reported by Jonkers et al. [23], who found rhombohedral and cubic CaCO_3_ crystals in concrete cracks repaired by Bacillus. Similarly, Xu and Yao et al. [25] reported the formation of compact CaCO_3_ precipitation layers at crack interfaces in Bacillus-based self-healing concrete systems. When combined with XRD analysis, the diffraction peaks at 29.4° and 27.4° correspond to calcite and vaterite, respectively. Furthermore, the TG/DTG results show a mass loss of 13–15% in the 600–800 °C range, which is higher than the 8% observed in the control group. This indicates a higher content of CaCO_3_ in the samples containing microbial self-healing materials. Overall, the combined SEM, XRD, and TG/DTG results confirm the formation of microbial-induced calcium carbonate precipitation within the cracks, which contributes to the healing of cracks and pores in concrete.

## 4. Conclusions

In this paper, the influence of microbial self-healing materials on the crack resistance and repair effect of concrete are studied, and the main conclusions are as follows:

(1) The incorporation of microbial self-healing materials into concrete effectively delays the initial cracking time. Specifically, compared with the blank group (90 min), the initial cracking time was increased by 38.9%, 11.1%, and 66.7% when adding Component A, Component B, and the combined use of Component A and Component B, respectively. When only Component A dosage was considered, the initial cracking time of each test group (10%, 20%, and 30%) was prolonged by 50%, 65%, and 70%, respectively, compared with the blank group without Component A (0%).
(2) The incorporation of microbial self-healing materials may slightly reduce the early-age crack resistance of concrete, but it does not adversely affect its later crack-healing performance. After 28 d of water spraying and coating curing, the total crack area of concrete decreased significantly compared with that at the early age (1 d). With the increasing dosage of Component A (0%, 10%, 20%, and 30%), the total crack area per unit surface area decreased by 12.2%, 21.9%, 22.7%, and 31.8%, respectively, compared with the early stage. Therefore, microbial self-healing materials exhibit a dual effect on early-age crack behavior in concrete, characterized by “delayed crack initiation (beneficial)” and “enhanced crack development (detrimental)”, which together define their early-age performance.
(3) Through X-ray diffraction (XRD), thermal analysis, and morphological characterization, it was confirmed that microbial mineralization products, including calcite and vaterite, were present on the concrete crack surfaces. With the increasing dosage of the microbial self-healing material (0%, 10%, 20%, and 30%), the content of these mineralization products increased from 10.95% to 15.23%.

## Figures and Tables

**Table 1:** Chemical composition of cement.

CaO	SiO_2_	Al_2_O_3_	SO_3_	Fe_2_O_3_	MgO
58.70%	23.24%	6.72%	3.28%	3.22%	3.03%

**Table 2:** Phase composition of cement.

C_3_S	C_2_S	C_3_A	C_4_AF
54.0%	19.0%	8.0%	10.0%

**Table 3:** Chemical composition of fly ash.

SiO_2_	Al_2_O_3_	Fe_2_O_3_	CaO	SO_3_
51.8%	27.9%	6.8%	3.1%	1.6%

**Table 4:** Concrete mix ratios with different amounts of microbial solution.

No.	Cement/kg	Fly Ash/kg	Fine Aggregate/kg	Coarse Aggregate/kg (5–25 mm)	Water Reducer/kg	Water/kg	Component a Bacterial Solution/kg	Component b Calcium Acetate/kg Urea/kg	
GA	17.8	1.6	25.6	46.4	0.21	6.0	—	—	—
GB	17.8	1.6	25.6	46.4	0.21	5.4	0.6	—	—
GC	17.8	1.6	25.6	46.4	0.21	6.0	—	0.4	0.14
GD	17.8	1.6	25.6	46.4	0.21	5.4	0.6	0.4	0.14
GE	17.8	1.6	25.6	46.4	0.21	4.8	1.2	0.4	0.14
GF	17.8	1.6	25.6	46.4	0.21	4.2	1.8	0.4	0.14

**Table 5:** Cracking condition of 1 d slab with different dosages of concrete.

No.	Wind Speed m/s	Initial Crack Time /min	Crack Size/mm		Number of Cracks/Article
			Maximum Crack Width	Maximum Crack Length	
GA	5.0 ± 0.5	90 ± 5	0.53	211	28
GB	5.0 ± 0.5	125 ± 5	0.50	189	40
GC	5.0 ± 0.5	100 ± 5	0.63	221	37
GD	5.0 ± 0.5	150 ± 5	0.60	211	39
GE	5.0 ± 0.5	165 ± 5	0.68	201	44
GF	5.0 ± 0.5	170 ± 5	0.58	164	49

## Data Availability

The original contributions presented in this study are included in the article. Further inquiries can be directed to the corresponding authors.
